# Supplementary material for: MetaRibo-Seq measures translation in microbiomes
Source: Nat Commun. 2020 Jun 29;11:3268. doi: 10.1038/s41467-020-17081-z (PMC7324362; doi:10.1038/s41467-020-17081-z)
Supplement: Supplementary file 10 — Supplementary Data 7 [file 41467_2020_17081_MOESM10_ESM.zip › File2/Confidence_VeryHigh_Taxonomy/345471_out.krona.html]

Javascript must be enabled to view this page.

members
magnitude
magnitudeUnassigned
count
unassigned
taxon
rank

345471\_out

6

2
6
superkingdom

976
6
phylum

6
class
200643

171549
6
order

815
6
family

816
genus
6

2

SRS011134\_contig\_number\_5985SRS022609\_contig\_number\_42464
species
162156

4

SRS015663\_contig\_number\_7365SRS016335\_contig\_number\_18319SRS047014\_contig\_number\_5454SRS142890\_contig\_number\_39816
species
338188
